# The infectious synapse formed between mature dendritic cells and CD4^+^ T cells is independent of the presence of the HIV-1 envelope glycoprotein

**DOI:** 10.1186/1742-4690-10-42

**Published:** 2013-04-16

**Authors:** Maria T Rodriguez-Plata, Isabel Puigdomènech, Nuria Izquierdo-Useros, Maria C Puertas, Jorge Carrillo, Itziar Erkizia, Bonaventura Clotet, Julià Blanco, Javier Martinez-Picado

**Affiliations:** 1 AIDS Research Institute IrsiCaixa, Institut d’Investigació en Ciències de la Salut Germans Trias i Pujol, Universitat Autònoma de Barcelona, Badalona, 08916, Spain; 2 Institució Catalana de Recerca i Estudis Avançats (ICREA), Barcelona, Spain

**Keywords:** Infectious synapse, Virological synapse, Immunological synapse, Dendritic cell, CD4^+^ T cell, Cell-to-cell, HIV-1, *Trans*-infection, Transmission

## Abstract

**Background:**

Since cell-mediated infection of human immunodeficiency virus type 1 (HIV-1) is more efficient than cell-free infection, cell-to-cell propagation plays a crucial role in the pathogenesis of HIV-1 infection. Transmission of HIV-1 is enabled by two types of cellular contacts, namely, virological synapses between productively infected cells and uninfected target cells and infectious synapses between uninfected dendritic cells (DC) harboring HIV-1 and uninfected target cells. While virological synapses are driven by expression of the viral envelope glycoprotein on the cell surface, little is known about the role of envelope glycoprotein during contact between DC and T cells. We explored the contribution of HIV-1 envelope glycoprotein, adhesion molecules, and antigen recognition in the formation of conjugates comprising mature DC (mDC) and CD4^+^ T cells in order to further evaluate their role in mDC-mediated HIV-1 transmission at the immunological synapse.

**Results:**

Unlike virological synapse, HIV-1 did not modulate the formation of cell conjugates comprising mDC harboring HIV-1 and non-activated primary CD4^+^ T cells. Disruption of interactions between ICAM-1 and LFA-1, however, resulted in a 60% decrease in mDC-CD4^+^ T-cell conjugate formation and, consequently, in a significant reduction of mDC-mediated HIV-1 transmission to non-activated primary CD4^+^ T cells (p < 0.05). Antigen recognition or sustained MHC-TcR interaction did not enhance conjugate formation, but significantly boosted productive mDC-mediated transmission of HIV-1 (p < 0.05) by increasing T-cell activation and proliferation.

**Conclusions:**

Formation of the infectious synapse is independent of the presence of the HIV-1 envelope glycoprotein, although it does require an interaction between ICAM-1 and LFA-1. This interaction is the main driving force behind the formation of mDC-CD4^+^ T-cell conjugates and enables transmission of HIV-1 to CD4^+^ T cells. Moreover, antigen recognition boosts HIV-1 replication without affecting the frequency of cellular conjugates. Our results suggest a determinant role for immune activation driven by mDC-CD4^+^ T-cell contacts in viral dissemination and that this activation likely contributes to the pathogenesis of HIV-1 infection.

## Background

Dendritic cells (DC) play a key role in the initiation of primary T-cell-mediated immune responses *in vivo*, as they are the most potent antigen-presenting cells (APC) in the immune system, especially upon maturation [1]. Interactions between DC and T cells can occur in the presence or absence of cognate antigen, both leading to various T-cell responses [2-4]. The initial stages of DC-CD4^+^ T-cell conjugate formation are antigen-independent, suggesting that DC-T cell adhesion precedes antigen recognition [3,4]. Although antigen-independent interactions do not induce full activation of T cells—as occurs in antigen-dependent interactions—they do maintain the homeostasis of naïve T cells [2,3,5]. Adaptive immune responses are initiated by the interaction of the appropriate peptide-major histocompatibility complex (pMHC) molecular complex on the APC with the T-cell receptor (TcR), which constitutes the basis of the immunological synapse. The immunological synapse provides sustained T-cell signaling, leading to T-cell priming and TcR downregulation [6,7]. This specialized synapse consists of a highly stable and organized area of contact between the APC and the T cell, where the pMHC-TcR interaction, adhesion molecules, and co-stimulatory molecules play a major role [8,9].

Human immunodeficiency virus type 1 (HIV-1) preferentially infects CD4^+^ T lymphocytes through the interaction of the gp120 subunit of the viral envelope glycoprotein (Env) with the CD4 receptor and an appropriate co-receptor on the surface of the T cell [10]. The classical model of HIV-1 spread involves binding of cell-free virions to permissive target cells. However, HIV-1 can subvert existing cellular communication pathways to enhance and potentiate viral propagation [11-14]. Direct cell-to-cell transmission provides advantages for retroviruses, by allowing them to obviate the dilution in the extracellular space that limits viral attachment [15,16]. Cell-to-cell propagation could play a crucial role in the pathogenesis of HIV-1, since cell-mediated HIV-1 transmission is estimated to be 100 to 1,000 times more efficient than cell-free virus spread [17]. Indeed, mathematical models have predicted that cell-to-cell transmission of HIV-1 accounts for ~90% of new infection events in lymphoid tissue [18].

Cell-mediated infection of a target cell by HIV-1 can occur via the formation of virological synapses with productively infected cells [11,19,20] or through infectious synapses with non-infected DC harboring HIV-1 [12,21]. Although the virological synapse has been extensively studied in the context of T cell-T cell viral transmission [11,20,22], infected immature DC or macrophages can act alternatively as effector cells [23-26], while uninfected DC can also act as target cells [25,27]. Cell adhesion in virological synapses is driven by the engagement of the CD4 molecule on the target cell with the viral Env on the surface of HIV-1-infected donor cells, which increases conjugate formation and favors viral transfer [11,20,22,27]. Other authors have also suggested a role for leukocyte function-associated antigen (LFA-1), intercellular adhesion molecule 1 (ICAM-1), and intercellular adhesion molecule 3 (ICAM-3) in this kind of cell contacts [28]. Unlike the virological synapse, the infectious synapse does not rely on productive infection of DC, but also allows for viral transmission to target CD4^+^ T cells [29]. In this mechanism, known as *trans*-infection, DC capture HIV-1 and confine viruses in a non-degrading compartment, thus preserving their infectivity. After interaction with CD4^+^ T cells, mature DC (mDC) release the virions on the contact zone, which enables infection of the target cell [12,30-32]. Furthermore, lipopolysaccharide (LPS) concentrations, which are significantly increased in chronically HIV-1-infected individuals as a result of microbial translocation [33], and other activation signals [34,35] mature DC and trigger their co-stimulatory capacity and their efficiency to capture and *trans-*infect HIV-1 [31,32,34,36,37]. Although the involvement of the DC-specific ICAM-3-grabbing non-integrin (DC-SIGN) [29,38], ICAM-1, and LFA-1 [34,37,39,40] is well documented, the cell-surface molecules that contribute to the formation of the infectious synapses have not been fully identified. In addition, little is known about the role of HIV-1 Env during the cell-to-cell interaction at the infectious synapse.

Interestingly, the infectious synapse was first characterized in mDC-CD4^+^ T-cell conjugates in the absence of antigen-specific signaling [12]. Upon contact between DC and T cells, the CD4, CCR5, and CXCR4 receptors on the T cell and the HIV-1 harbored by the DC are recruited to the contact interface, thus facilitating HIV-1 transmission [12]. Since DC are present in peripheral tissue, such as mucosa, they are thought to be among the first cells to encounter HIV-1 during sexual transmission [41-43]. Consequently, DC scavenge for HIV-1 in peripheral tissue before moving on to lymphoid tissue, where HIV-1 gains access to CD4^+^ T lymphocytes, the main targets of infection [35,43-45]. Thus, DC-T cell interactions, which are crucial for the generation of specific immune responses, can be hijacked by HIV-1 to bypass immune control and amplify viral replication [21,31,46,47].

In this study, we explored the contribution of HIV-1 Env during conjugate formation at the infectious synapse and analyzed its role in combination with adhesion molecules in the context of antigen presentation. Our data showed that, in contrast with the virological synapse, HIV-1 did not modulate the formation of the infectious synapse between mDC harboring HIV-1 and uninfected CD4^+^ T cells. On the contrary, the main driving force behind formation of mDC-CD4^+^ T-cell conjugates and HIV-1 *trans-*infection of CD4^+^ T cells was the interaction between ICAM-1 and LFA-1. In addition, antigen recognition or sustained MHC-TcR interaction did not enhance conjugate formation, but significantly boosted productive DC-mediated HIV-1 *trans-*infection. Consistently, antigen recognition also significantly increased T-cell activation and proliferation after conjugation with mDC. Our results suggest a determinant role of contact between mDC and CD4^+^ T cells in immune activation and viral dissemination, which likely contribute to the pathogenesis of HIV-1 infection.

## Results

### mDC-CD4^+^ T-cell conjugate formation is HIV-1-independent

**Figure 1:**
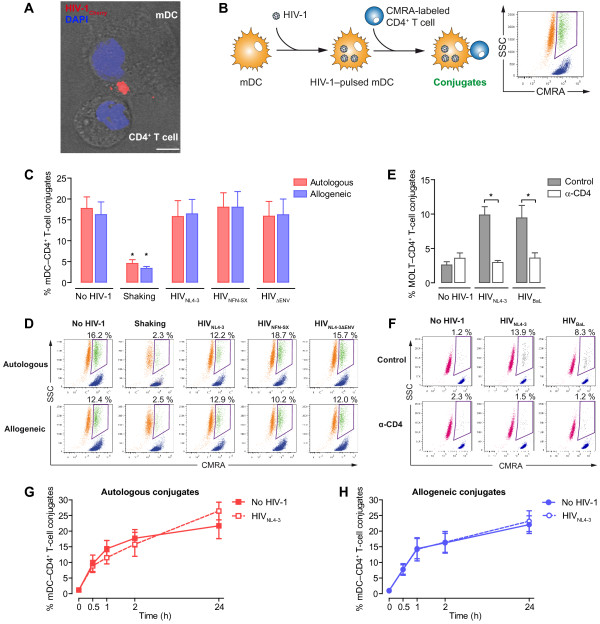
**Conjugate formation between mDC harboring HIV-1 and primary CD4**^**+ **^**T cells is independent of the presence of HIV-1. A.** Confocal microscopy analysis of a HIV-1-pulsed mDC-CD4^+^ T-cell synapse, showing the polarization of the HIV-1-containing intracellular compartment in mDC to the contact zone. Merge of the bright field and the fluorescence of an x-y plane (scale bar: 5 μm). **B.** Experimental procedure for quantification of cellular conjugates between mDC and CMRA-labeled CD4^+^ T cells by flow cytometry. Events with similar morphology to mDC (SSC) but simultaneously positive for the CMRA were considered stable cellular conjugates between mDC and primary CD4^+^ T cells (green). Events corresponding to mDC are in orange, and CMRA-labeled CD4^+^ T cells are in blue. **C.** Comparative quantification of cellular conjugates between mDC and non-activated primary CD4^+^ T cells in the presence or absence of HIV-1 after 2 hours of autologous (red) or allogeneic (blue) co-culture. **D.** Representative experiment of quantification of cellular conjugates between mDC preloaded with different HIV-1 strains and non-activated CMRA-labeled CD4^+^ T cells by flow cytometry; cellular conjugates (green), mDC (orange), and CMRA-labeled CD4^+^ T cells (blue). **E.** Comparative quantification of cellular conjugates between MOLT cell lines chronically infected with X4- (MOLT_NL4-3_) or R5-tropic (MOLT_BaL_) HIV-1 and non-activated primary CD4^+^ T cells after 2 hours of co-culture. **F.** Representative experiment of quantification of cellular conjugates between HIV-1-infected MOLT cells and non-activated CMRA-labeled CD4^+^ T cells by flow cytometry; cellular conjugates (black), MOLT cells (red), and CMRA-labeled CD4^+^ T (blue). **G**. and **H.** Kinetics of conjugate formation in autologous (**G**) or allogeneic (**H**) mDC-CD4^+^ T-cell co-cultures in the presence or absence of HIV-1. Asterisks indicate significant differences compared with co-cultures in the absence of HIV-1 (p < 0.05). Data are expressed as mean and SEM from three independent experiments including cells from six different donors.

The interaction between the viral Env on the surface of the HIV-1-infected cell and its primary receptor, CD4, on the target cell is one of the determinants in the formation of the virological synapse, as we and other authors have reported [11,20,22,27]. Moreover, the dependence of the virological synapse on Env increases the frequency of stable cell conjugates comprising HIV-1-infected and uninfected T cells, thus facilitating the transfer of virus to target cells [11,20,22,27]. However, the role of HIV-1 Env in the cell-to-cell interaction at the infectious synapse between uninfected DC harboring HIV-1 and target CD4^+^ T lymphocytes has not yet been addressed. Since mDC capture large amounts of HIV-1 particles [31,34], and polarizes the HIV-1-containing intracellular compartment to the contact zone with target cells (Figure 1A) [12,32,48], the Env of trapped virions could guide the formation of mDC-CD4^+^ T-cell conjugates.

In order to evaluate whether the presence of HIV-1 drives the formation of conjugates comprising mDC harboring HIV-1 and uninfected CD4^+^ T cells, we used a previously described flow cytometry method [20] to quantify cellular conjugates between CMRA-labeled uninfected CD4^+^ T cells and mDC pulsed or not with viral particles. This approach enabled us to identify cellular conjugates as those events with a similar morphology to mDC (defined by SSC values) and high levels of fluorescence from the CMRA-labeled CD4^+^ T cells (Figure 1B). In addition, we also assessed the contribution of antigen recognition during conjugate formation. To that end, we used autologous and allogeneic co-cultures of mDC and non-activated CD4^+^ T cells in the presence or absence of HIV-1 to evaluate antigen-independent and antigen-dependent interactions, respectively. Although alloreactivity does not involve an antigen-specific response, allorecognition is characterized by a stable MHC-TcR interaction that elicits an exceptionally vigorous T-cell response [49]. Besides, as many as 1–10% of T lymphocytes can respond to allogeneic MHC molecules [50,51]; this frequency is several orders of magnitude above the frequency of specific T cells for any single foreign antigen presented by self-MHC [49,52].

Unlike the virological synapse, the presence of HIV-1 did not increase the formation of conjugates between mDC harboring HIV-1 and non-activated primary CD4^+^ T cells. Thus, unpulsed mDC or mDC pulsed with X4-tropic HIV_NL4-3_, R5-tropic HIV_NFN-SX_, or Env-deficient HIV_NL4-3ΔENV_ displayed the same percentage of cellular conjugates (within a range of 15.8–19.9%) with non-activated CD4^+^ T cells (Figure 1C and D), revealing that viral tropism or even the HIV-1 Env itself had no effect on the formation of mDC-CD4^+^ T-cell conjugates. As previously described [53], limitation of cell contacts by continuous shaking significantly inhibited the formation of cellular conjugates (within a range of 3.7–5.3%) (p < 0.05) (Figure 1C and D). Neither the presence of HIV-1 nor the capture and internalization of virus by mDC impacted the conjugate formation with non-activated CD4^+^ T cells. This finding contrasts somewhat with the T cell-T cell virological synapses formed between productively infected cells and uninfected primary CD4^+^ T cells [20,22]. We analyzed this by using uninfected primary CD4^+^ T cells in co-culture with the T-lymphoblastoid MOLT cell lines chronically infected with X4-tropic (MOLT_NL4-3_) HIV-1 or R5-tropic (MOLT_BaL_) HIV-1 and observed a higher frequency of cellular conjugates with the HIV-1-infected T cells (both 9.2%) than with uninfected MOLT cells (2.6%) (Figure 1E and F). In addition, the virological synapses were inhibited by blocking gp120 binding to CD4 using the α-CD4 Leu3a monoclonal antibody (mAb), reaching levels similar to those observed between uninfected MOLT cells and primary CD4^+^ T cells (p < 0.5, Figure 1E and F).

Surprisingly, autologous and allogeneic co-cultures between mDC and CD4^+^ T cells displayed a comparable percentage of cell conjugates (Figure 1C and D), indicating that sustained MHC-TcR recognition at the contact zone did not affect the number of conjugates detected. In addition, the frequency of mDC-CD4^+^ T-cell conjugates increased over time in both autologous and allogeneic co-cultures (Figure 1G and H), regardless of the presence or absence of HIV-1 (Figure 1G and H). These findings indicated that, contrary to the virological synapse, the presence of HIV-1 did not modulate the formation of conjugates comprising mDC harboring HIV-1 and uninfected primary CD4^+^ T cells.

### Blocking of ICAM-1 and LFA-1 impairs the formation of mDC-CD4^+^ T-cell conjugates

**Figure 2:**
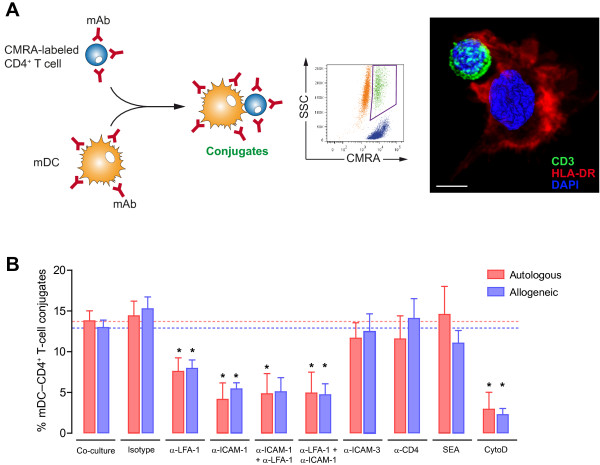
**Blocking of ICAM-1 and LFA-1 impairs conjugate formation between mDC and primary CD4**^**+ **^**T cells. A.***Left.* Experimental procedure for quantification of cell conjugates between mDC and CMRA-labeled CD4^+^ T cells in the presence or absence of several reagents. Cell conjugates were analyzed by flow cytometry as in Figure 1B. *Right.* Phenotype of mDC-CD4^+^ T cell contact by confocal microscopy. HLA-DR and CD3 molecules were stained to unequivocally identify the mDC and the CD4^+^ T cell, respectively. Confocal Z-stacks were acquired every 0.25 μm steps and processed with Volocity 6.1 software (improvision, PerkinElmer) using the maximum fluorescent intensity projection for HLA-DR and CD3 stainings and the isosurface modeling for DAPI-stained nucleus (scale bar: 5 μm). **B.** Comparative quantification of cellular conjugates after 2 hours of autologous (red bars) or allogeneic co-cultures (blue bars) between mDC and non-activated CD4^+^ T cells. Blocking of LFA-1 and ICAM-1 significantly inhibited the formation of autologous and allogeneic conjugates. Both autologous and allogeneic conjugates were equally affected by the blocking reagents. No differences were detected between autologous and allogeneic co-cultures. The α-ICAM-1 + α-LFA-1 condition represents separate pre-incubation of mDC with α-ICAM-1 mAb and CD4^+^ T cells with α-LFA-1 mAb, before launching co-culture, while the α-LFA-1 + α-ICAM-1 condition designates separate pre-incubation of mDC with α-LFA-1 mAb and CD4^+^ T cells with α-ICAM-1 mAb. Asterisks indicate significant differences compared with negative controls (p < 0.05); mAb conditions were compared with an isotype control, whereas SEA and cytochalasin D were compared with medium. Data are expressed as mean and SEM from at least three independent experiments including cells from at least six different donors.

Since we observed that HIV-1 and antigen recognition appeared to exercise albeit minimal control over the interaction between mDC and CD4^+^ T cells, we analyzed the role of adhesion molecules during formation of mDC-CD4^+^ T-cell conjugates (Figure 2A). Engagement of ICAM-1 and LFA-1 is considered essential for the formation of DC-T cell immunological synapses [54] and the infiltration of lymphocytes into sites of inflammation [55]. Therefore, we specifically evaluated the role of these adhesion molecules in the formation of mDC-CD4^+^ T-cell conjugates by using blocking mAb against these adhesion molecules. We observed that treatment of both mDC and CD4^+^ T cells with α-LFA-1 (mAb 68.5A5) reduced the frequency of cellular conjugates by 45% (p < 0.05, Figure 2B), whereas blocking of ICAM-1 with mAb RM3A5 inhibited the formation of autologous and allogeneic conjugates by 60% (p < 0.05, Figure 2B). However, we did not observe a synergistic effect when ICAM-1 on mDC and LFA-1 on CD4^+^ T cells were blocked, and vice versa (p < 0.05, Figure 2B). Moreover, the addition of blocking α-ICAM-3 mAb to both mDC and CD4^+^ T cells failed to inhibit the formation of cellular conjugates (Figure 2B). Despite the relevance of the CD4 receptor in the formation of the virological synapse (Figure 1E) [11,20,22], the blockade of the CD4 molecule with mAb Leu3a did not have any significant impact on the formation of mDC-CD4^+^ T-cell conjugates (Figure 2B).

Engagement of CD4 by Env at the virological synapse between infected and uninfected CD4^+^ T cells triggers actin-dependent recruitment of HIV-1 receptors and adhesion molecules to the contact interface, thus stabilizing the adhesive interactions and enabling the final transfer of HIV-1 to the target cell [11,22,56]. Consequently, we analyzed whether the cytoskeleton was necessary for the establishment of the mDC-T-cell interaction. Addition of cytochalasin D effectively blocked the formation of mDC-CD4^+^ T-cell conjugates (p < 0.05) (Figure 2B), indicating that this process requires an active actin cytoskeleton to rearrange receptors towards the interface of the mDC-T-cell contact. Conversely, the presence of bacterial superantigen (SEA) in the co-culture did not increase the percentage of cellular conjugates (Figure 2B), probably because the SEA induced long-lasting interactions between mDC and T cells, thus enabling the formation of more stable conjugates and the subsequent functional maturation of the immunological synapse [57]. As shown in Figure 1, both autologous and allogeneic co-cultures yielded similar percentages of cellular conjugates and were equally susceptible to the blocking reagents used in these experiments (Figure 2B), thus confirming that neither antigen recognition nor sustained MHC-TcR interaction alone enhanced conjugate formation. Taken together, these data suggest that the adhesion molecules ICAM-1 and LFA-1 are the main driving force in modulating the formation of mDC-CD4^+^ T-cell conjugates and could play a key role in transmission of HIV-1 across the infectious synapse.

### mDC-mediated HIV-1 *trans-*infection of primary CD4^+^ T cells is dependent on the interaction between ICAM-1 and LFA-1 and is enhanced by antigen recognition

**Figure 3:**
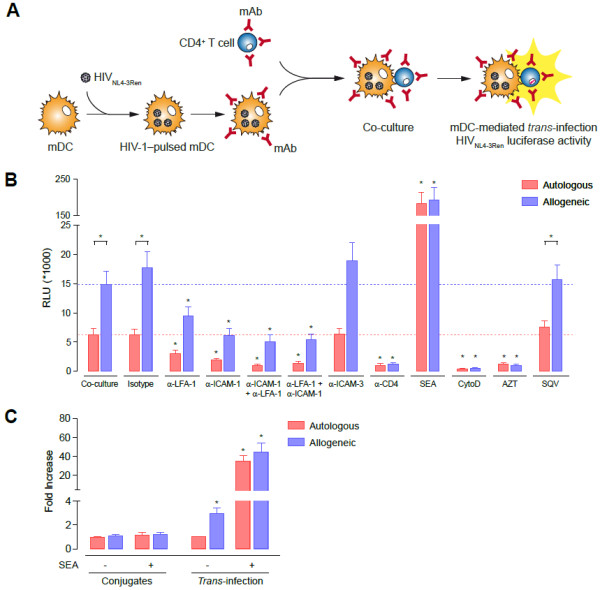
**mDC-mediated HIV-1 *trans-*infection to primary CD4**^**+ **^**T cells is dependent on the interaction between ICAM-1-LFA-1 and is enhanced by antigen recognition. A.** Experimental procedure for quantification of mDC-mediated *trans-*infection of the infectious reporter virus HIV_NL4-3Ren_ to non-activated CD4^+^ T cells in the presence of several reagents. **B.** Comparative HIV-1 mDC-mediated *trans-*infection of non-activated CD4^+^ T cells in autologous or allogeneic co-cultures in the presence of several reagents. Allogeneic co-cultures (blue bars) led to a three-fold increase in productive HIV-1 replication compared with autologous co-cultures (red bars) (p < 0.05). Blocking ICAM-1 and LFA-1 with mAb significantly inhibited the mDC-mediated HIV-1 *trans*-infection. The α-ICAM-1 + α-LFA-1 condition represents separate pre-incubation of mDC with α-ICAM-1 mAb and CD4^+^ T cells with α-LFA-1 mAb, before launching co-culture, whereas the α-LFA-1 + α-ICAM-1 condition designates separate pre-incubation of mDC with α-LFA-1 mAb and CD4^+^ T cells with α-ICAM-1 mAb. Asterisks indicate significant differences compared with negative controls (p < 0.05); mAb conditions were compared with the isotype control, whereas AZT, SQV, SEA and cytochalasin D were compared with medium. Data are expressed as mean and SEM from three independent experiments including cells from at least six different donors. RLU, relative light units. **C.** Fold increase of conjugate formation and mDC-mediated HIV-1 *trans-*infection in autologous and allogeneic mDC-CD4^+^ T cell co-cultures in the presence or absence of SEA. All conditions were compared to autologous co-cultures without SEA, shown as unity in the plot.

Once we confirmed that ICAM-1 and LFA-1 and not HIV-1 or antigen recognition modulate the formation of mDC-CD4^+^ T-cell conjugates, we investigated the role of these factors in mDC-mediated HIV-1 *trans*-infection of primary CD4^+^ T cells. We performed both autologous and allogeneic co-cultures of mDC pulsed with the reporter virus HIV_NL4-3Ren_ and non-activated primary CD4^+^ T cells in the presence of several blocking reagents (Figure 3A). In this set of experiments, a co-culture performed with the protease inhibitor saquinavir (SQV) allowed us to confirm net *trans*-infection, thus avoiding re-infection events between T cells (Figure 3), while a control co-culture with azidothymidine (AZT), which completely blocked luciferase activity, confirmed productive HIV-1 replication in co-cultures.

Allogeneic conjugates between HIV-1-pulsed mDC and non-activated primary CD4^+^ T cells led to a three-fold greater increase in viral replication than autologous co-cultures (p < 0.05, Figure 3B and C). This observation suggests a role for antigen recognition in HIV-1 replication in CD4^+^ T lymphocytes, probably because of an increase in cell activation mediated by contact with mDC. Consistent with this hypothesis, we observed a dramatic increase in HIV-1 replication as a result of mDC-mediated *trans*-infection when MHC-TcR interactions were stabilized with SEA (p < 0.05, Figure 3B and C), which corresponds to one viral replication cycle (Additional file 1 A). Interestingly, direct infection of non-activated primary CD4^+^ T cells by free HIV-1 was almost null, even under α-CD3 (mAb OKT3) or SEA activation conditions (Additional file 1 B). This could be explained because SEA needs to simultaneously bind to MHC class II molecule on the APC and to the TcR on the CD4^+^ T cell to lead a non-specific stimulation of T lymphocytes [58,59]. Meanwhile, activation of T cells via CD3 needs costimulatory signaling provided by APC. Therefore, the activation and subsequent HIV-1 infection of CD4^+^ T cell cultured with SEA and α-CD3 depends on the presence of an APC, in this case mDC, which provides MHC or costimulatory signaling. Consequently, the increase in the mDC-mediated *trans-*infection, either in autologous or allogeneic co-cultures, in α-CD3 and SEA conditions is dependent of the presence of mDC.

Some reports have already illustrated the importance of the interaction between ICAM-1 and LFA-1 in facilitating transmission of HIV-1 between DC and target cells [34,37,40]. Furthermore, ICAM-1 is the main force driving formation of mDC-CD4^+^ T-cell conjugates (Figure 2). As expected, blockade of ICAM-1 with the mAb RM3A5 not only inhibited the formation of mDC-CD4^+^ T-cell conjugates (Figure 2B), but also impaired the mDC-mediated *trans*-infection of HIV-1 to CD4^+^ T lymphocytes by 60% to 70% (p < 0.05, Figure 3B). Productive infection of CD4^+^ T cells was also significantly abrogated by 45 to 50% when cells were treated with the mAb α-LFA-1 68.5A5 (p < 0.05, Figure 3B). In addition, when both ICAM-1 and LFA-1 were simultaneously blocked, inhibition of mDC-mediated *trans-*infection of HIV-1 to primary CD4^+^ T cells reached 75% to 90% in both autologous and allogeneic co-cultures (p < 0.05, Figure 3B). Consequently, blocking of ICAM-1-LFA-1 interactions between mDC and non-activated primary CD4^+^ T cells substantially reduced the productive mDC-mediated HIV-1 *trans*-infection of CD4^+^ T cells. In addition, treatment with α-ICAM-3 did not reduce viral replication in target cells (Figure 3B), thus confirming ICAM-3-independent mDC-mediated transmission of HIV-1 to primary CD4^+^ T cells [37]. On the other hand, although blocking the CD4 receptor did not affect the formation of cellular conjugates (Figure 2B), the addition of α-CD4 mAb Leu3a successfully inhibited productive infection of CD4^+^ T cells through mDC-mediated *trans-*infection of HIV-1 by hampering binding of HIV-1 Env to the CD4 molecule in target cells (p < 0.05, Figure 3B). In addition, we confirmed that DC-mediated transmission of HIV-1 to primary CD4^+^ T cells was also dependent on the integrity of the actin cytoskeleton, since cytochalasin D impaired recruitment of receptors to the infectious synapse (p < 0.05, Figure 3B). Taken together, these findings indicate that the interactions between ICAM-1 and LFA-1 in mDC and primary CD4^+^ T cells, as well as a functional actin cytoskeleton, were necessary for efficient mDC-mediated HIV-1 *trans*-infection in an antigen-dependent and antigen-independent manner across the infectious synapse. Importantly, our data suggest that the role of antigen recognition in *trans*-infection of HIV-1 is restricted to late synaptic events, since cellular conjugation is unaffected by allogeneic or SEA stimulation, whereas productive infection of T cells is greatly enhanced (Figure 3C).

### Antigen-recognition, but not HIV-1, enhances CD4^+^ T-cell activation and proliferation

**Figure 4:**
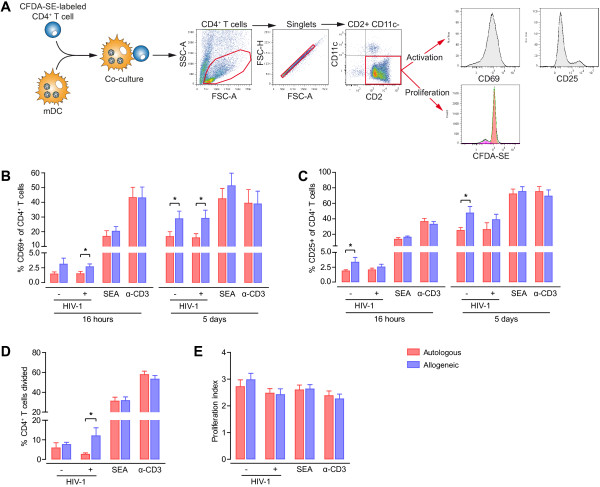
**CD4**^**+ **^**T-cell activation and proliferation is enhanced by antigen recognition but not by the presence of HIV-1. A.** Gating strategy for the analysis of CD4^+^ T-cell activation and proliferation after co-culture with mDC. CD69, CD25, and proliferation parameters were analyzed in cells within the CD2-positive, CD11c-negative singlet gate. As positive controls, CD4^+^ T cells alone or with mDC were cultured in the presence of SEA or α-CD3. **B.** CD69 expression in CD4^+^ T cells after co-culture for 16 hours or 5 days with autologous mDC (red bars) or allogeneic mDC (blue bars) in the presence or absence of HIV-1. Expression of CD69 surface marker was higher in CD4^+^ T cells co-cultured with allogeneic mDC. No differences were observed in the presence of HIV-1 compared with control co-cultures without virus. **C.** CD25 expression in CD4^+^ T cells after co-culture for 16 hours or 5 days with autologous mDC (red bars) or allogeneic mDC (blue bars) in the presence or absence of HIV-1. Stimulation with allogeneic mDC induced higher levels of CD25 expression in CD4^+^ T lymphocytes (p < 0.05). The presence of HIV-1 did not exert a detectable effect on CD25 expression. **D.** and **E.** Percentage of CD4^+^ T cells divided (**D**) and proliferation index of CD4^+^ T cells (number of cell divisions/number of divided cells) (**E**) after co-culture for 5 days with autologous mDC (red bars) or allogeneic mDC (blue bars) in the presence or absence of HIV-1. Allogeneic co-cultures exhibited more CD4^+^ T-cell proliferation than autologous co-cultures, although no significant differences were observed with regard to the presence or absence of HIV-1. Positive controls consisting of co-cultures with SEA or α-CD3 are shown. Data are expressed as mean and SEM from three independent experiments including cells from at least six different donors. Asterisks denote significant differences (p < 0.05).

HIV-1 replication is highly dependent on the activation status of target cells, since highly activated T lymphocytes are the main target for viral spread and consequent cell destruction [60,61]. To elucidate the level of activation in primary CD4^+^ T cells after antigen-dependent and antigen-independent contacts with mDC, we evaluated surface expression of the activation markers CD69 and CD25 after 16 hours and 5 days of autologous and allogeneic co-cultures. We also monitored the T-cell proliferation induced after 5 days of co-culture using a flow cytometry assay based on carboxyfluorescein diacetate succinimidyl ester (CFDA-SE). Simultaneously, we assessed the effect on these parameters induced by the presence of HIV-1 in the co-culture. CD4^+^ T cells were distinguished from mDC in co-cultures by gating cellular singlets and selecting the CD2-positive, CD11c-negative population; surface activation markers and proliferation were then analyzed within lymphocytes (Figure 4A). As positive controls, SEA or mAb α-CD3 OKT3 were added to mDC-CD4^+^ T-cell co-cultures.

Expression levels of CD69 and CD25 increased over time in CD4^+^ T cells cultured with allogeneic mDC, as compared with autologous co-cultures (p < 0.05, Figure 4B and C). Consequently, the antigen-dependent HIV-1 infection and virus replication we observed in Figure 3B could be associated with the higher immune activation of CD4^+^ T cells mediated by allogeneic mDC. Interestingly, similar levels of CD69 and CD25 expression were detected in CD4^+^ T cells cultured with HIV-1-pulsed or -unpulsed mDC, both in autologous and in allogeneic co-cultures (Figure 4B and C). This finding contrasts with the results of other studies that reveal a substantial Nef-dependent increase in CD69 expression in T cells co-cultured with HIV-1-infected immature DC [62]. Since the HIV_NL4-3Ren_ used in our experiments lacks the gene *nef*, the CD4^+^ T-cell activation in our experimental setting should be driven by the contacts with mDC. Furthermore, in our experiments we used LPS-matured DC expressing high levels of MHC and co-stimulatory molecules at the cell membrane [36], with competence to stimulate T cells and poor ability to support viral replication [46].

After 5 days of co-culture, we examined the T-cell proliferation induced by the autologous and allogeneic contacts with mDC in the presence or absence of HIV-1 by analyzing the percentage of the CD4^+^ T cells divided (Figure 4D) and the average number of divisions in the responding cells (proliferation index, Figure 4E). More CD4^+^ T cells from allogeneic co-cultures than from autologous co-cultures divided at least once in the presence of HIV-1 (p < 0.05, Figure 4D), although this trend was also observed in the absence of HIV-1. However, the responding T lymphocytes from autologous and allogeneic co-cultures underwent the same number of proliferation cycles, independently of HIV-1 (Figure 4E). Consequently, the mixed lymphocyte reaction (MLR) between allogeneic mDC and CD4^+^ T cells activated more lymphocytes to proliferate, but responding cells supported the same number of cell divisions. These data strongly suggest that immune activation of T cells mediated by mDC can provide an environment to facilitate HIV-1 transmission and replication. A more sustained MHC-TcR interaction due to antigen-dependent contacts between APC and T cells allows higher lymphocyte activation and, consequently, increased susceptibility to infection of target cells. Therefore, this observation is consistent with the findings that antigen-specific CD4^+^ T cells are preferentially infected by HIV-1 *in vivo*, resulting in depletion of responding CD4^+^ T lymphocytes and loss of immunological control of HIV-1 replication [63-65].

## Discussion

Immune cells communicate with each other through cell-to-cell contacts. Viruses such as HIV-1 can take advantage of these contacts to amplify viral infection. By hijacking the existing pathways of cell-to-cell communication, HIV-1 can evade certain stages of the humoral immune response [16] and reach the final target of infection, namely, CD4^+^ T lymphocytes. It has been predicted that the vast majority of HIV-1-infected cells in lymphoid tissue are infected through cell-to-cell transmission [18], since cell-mediated HIV-1 infection is much more efficient than infection by cell-free virus [17]. DC, which are professional APC, are constantly scavenging for pathogens in peripheral tissue and interacting with other immune cells. In addition, mDC provide a perfect microenvironment for potentiating viral dissemination, because they can *trans-*infect HIV-1 by retaining and transmitting infectious virions without becoming infected [12,29-32,34,36]. Here, we characterized the molecular interactions at the infectious synapse between mDC harboring HIV-1 and non-activated primary CD4^+^ T cells where *trans-*infection takes place. We evaluated the contribution of HIV-1, adhesion molecules, and antigen recognition in conjugate formation, viral transmission, and cellular activation and proliferation.

**Figure 5:**
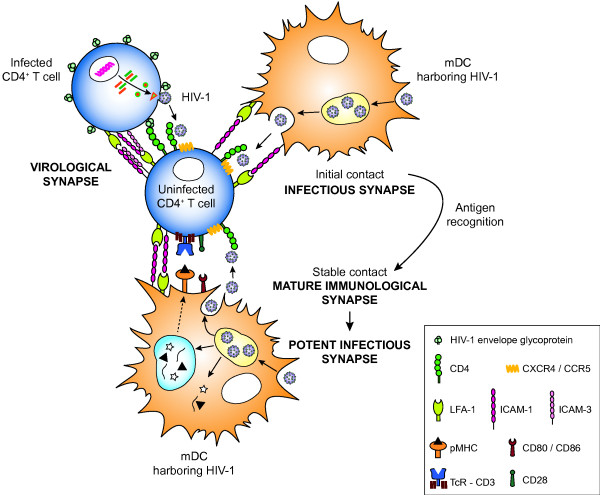
**Comparison between the formation of virological, infectious, and immunological synapses.** Different molecules are involved in the formation of these synapses, although they all enable infection of CD4^+^ T lymphocytes by HIV-1. The virological synapse relies on engagement between the Env on the surface of an HIV-1-infected cell with the CD4 molecule in the uninfected target cell and on adhesion molecules such as ICAM-1, LFA-1 and ICAM-3. On the other hand, the initial contact between a mDC and a CD4^+^ T cell is dependent on the adhesion molecules ICAM-1 and LFA-1. If cognate pMHC-TcR recognition occurs, contact between mDC and T cells stabilizes, thus constituting a mature immunological synapse. This antigen-specific signaling also involves co-stimulatory receptors and adhesion molecules and induces strong T-cell activation. When mDC harbor HIV-1, contacts between mDC and T cells would become infectious synapses, since HIV-1 could exploit the pre-existing cell-to-cell interactions to gain access to the target cells without modulating conjugate formation. Therefore, in the context of antigen-specific recognition between mDC harboring HIV-1 and T lymphocytes, immunological synapses would turn into potent infectious synapses by increasing the susceptibility of CD4^+^ T cells to productive HIV-1 infection. For simplicity, additional DC-T-cell interactions are not depicted.

We showed that, unlike virological synapses between productively HIV-1-infected cells and uninfected target cells [20,22,28], infectious synapses between DC harboring HIV-1 and uninfected CD4^+^ T cells did not rely on Env-CD4 interactions (Figure 5). Therefore, the measurement of cellular contacts by flow cytometry showed that uninfected CD4^+^ T cells established the same percentage of conjugates with mDC, independently of the presence or absence of HIV-1. Although the virological synapse between HIV-1-infected and uninfected T cells could be modulated by adhesion molecules [28], in the infectious synapse, adhesion molecules are important for the establishment of cellular contacts between mDC and CD4^+^ T lymphocytes. We showed that specific mAb against ICAM-1 and LFA-1 blocked mDC-CD4^+^ T-cell conjugation. This reduction in cell-to-cell adhesion also resulted in a marked decrease in the productive mDC-mediated HIV-1 *trans-*infection of primary CD4^+^ T cells (Figure 5). Although ICAM-3 contribute to the initial scanning of T lymphocytes and APC before antigen-specific recognition [66], blockade of ICAM-3 did not impact conjugate formation. Furthermore, specific mAb against ICAM-3 did not affect the transmission of HIV-1 between mDC and CD4^+^ T cells. This observation is consistent with the findings of other authors, who have demonstrated the relevance of ICAM-1 and LFA-1 [34,37], but not of ICAM-3 [37], in the DC-mediated transmission of HIV-1 across the infectious synapse. As with the virological synapse [11,56], and according to the results of other authors [67], we confirmed that the formation of the infectious synapse was an actin-dependent process. Remodeling of the actin cytoskeleton not only enables recruitment of receptors to the interface between mDC and CD4^+^ T cells to facilitate transmission of HIV-1 [12], but it also enables HIV-1 polarization and sac-like compartment formation in mDC upon viral capture [27,32].

Mature immunological synapses between mDC and T lymphocytes form as a result of robust cognate pMHC-TcR interaction, co-stimulatory receptors, and adhesion molecules, all of which leads to T-cell activation. Nevertheless, DC and T cells can also establish antigen-independent contacts [2], which, in the same way as the immunological synapses, are initiated by means of adhesion molecules[3,4]. Engagement between ICAM-1 and LFA-1 facilitates the pMHC-TcR interactions in immunological synapses [54], providing and consolidating positional stability to enhance T-cell sensitivity to antigen [68]. In the absence of antigen-specific recognition, these interactions result in the recruitment of molecules involved in the immunological synapse (eg, the HIV-1 receptors CD4, CXCR4, and CCR5 on the T cell) to the contact zone and in low levels of signaling in the T cell [2,12]. Moreover, in the presence of HIV-1, these contacts lead to polarization of the HIV-1-containing compartment in DC, thus facilitating viral transmission through the infectious synapse [12,21]. We confirmed that ICAM-1 and LFA-1 play a significant role in the infectious synapse, given that the blockade of these adhesion molecules equally affected autologous and allogeneic mDC-CD4^+^ T-cell co-cultures. However, the MLR in allogeneic co-cultures or the presence of SEA did not increase the percentage of cellular contacts compared to autologous co-cultures, indicating that MHC-TcR recognition did not increase the number of mDC-CD4^+^ T-cell conjugates. In contrast to the minor role in cell conjugation, the sustained MHC-TcR binding in allogeneic co-cultures and the presence of SEA boosted the productive DC-mediated HIV-1 *trans-*infection of CD4^+^ T cells through the infectious synapse by a mechanism that is strongly associated with the immune activation mediated by mDC through TcR signaling and co-stimulation.

Although the decisive factor for efficient HIV-1 transmission is co-receptor expression in the different T-cell subsets [69], the presence of DC enhances susceptibility to HIV-1 infection and replication in target cells [31,32,34,37,69]. The activation status and proliferation of T cells can have a considerable effect on the infectivity and replication of HIV-1. Antigen recognition, such as in alloantigen- or nominal antigen-specific interactions between DC and CD4^+^ T lymphocytes, induces full T-cell activation and proliferation through TcR and co-stimulatory signaling [2,54]. Nevertheless, contact between DC and CD4^+^ T cells in the absence of cognate antigen can also lead to a series of T-cell responses, such as weak proliferation and long-term survival, which are crucial for maintenance of homeostasis in the naïve T-cell pool *in vivo*[2]. In our experiments, mDC were able to *trans-*infect HIV-1 to autologous non-activated primary CD4^+^ T cells in the absence of nominal antigen, thus inducing lower levels of T-cell activation and proliferation. Consequently, HIV-1 could take advantage of labile mDC-CD4^+^ T-cell contacts, without antigen-specific recognition, to infect CD4^+^ T cells and potentially contribute to viral dissemination and HIV-1 latency. On the other hand, we have shown that allogeneic co-culture of mDC and CD4^+^ T lymphocytes induced higher levels of CD69 and CD25 expression and proliferation, independently of the presence of HIV-1, and that it resulted in higher viral *trans-*infection and replication in CD4^+^ T cells. Thus, stable cellular conjugates of DC and CD4^+^ T cells, such as those found in alloantigen or antigen-specific contacts, would enhance viral transmission across the synapse by increasing the susceptibility of target cells.

In summary (Figure 5), the initial stages of contact between mDC and CD4^+^ T cells are dependent on the adhesion molecules ICAM-1 and LFA-1. These interactions facilitate the recruitment of MHC, TcR, CD4, CXCR4, CCR5, and other molecules to the contact zone. Then, if cognate pMHC-TcR recognition occurs, cellular interactions consolidate into a mature immunological synapse, providing vigorous TcR and co-stimulatory signaling. When mDC harbor HIV-1, the virus could exploit these pre-existing cellular contacts to infect CD4^+^ T cells without perturbing the formation of cell conjugates. Consequently, either the contact between mDC and CD4^+^ T cells or the mature immunological synapse become infectious synapses. Furthermore, antigen-specific recognition would increase T-cell activation and, as a result, the susceptibility of CD4^+^ T cells to productive HIV-1 infection. Therefore, immunological synapses turn into potent infectious synapses, thus explaining why HIV-1 preferentially infects antigen-specific CD4^+^ T cells [63-65].

## Conclusions

The formation of infectious synapses between mDC and primary CD4^+^ T cells is independent of the presence of the HIV-1 Env. On the contrary, binding of ICAM-1 to LFA-1 is necessary for both the interaction between mDC and primary CD4^+^ T cells and efficient mDC-mediated HIV-1 *trans*-infection. Conversely, antigen recognition or sustained MHC-TcR interaction itself does not mediate cellular conjugation, but boosts productive DC-mediated HIV-1 *trans-*infection of primary CD4^+^ T lymphocytes by promoting T-cell activation and proliferation. Our results suggest a determinant role of immune activation driven by mDC-CD4^+^ T-cell contacts in viral dissemination, which likely contribute to the pathogenesis of HIV-1 infection.

## Methods

### Cells

Peripheral blood mononuclear cells (PBMC) from HIV-1-seronegative donors were purchased from the Banc de Sang i de Teixits (BST) after approval from the Ethics Committee of the University Hospital Germans Trias i Pujol. Purified monocyte populations were isolated with CD14^+^ positive selection magnetic beads (Miltenyi Biotec) and cultured with RPMI (Invitrogen) containing 10% fetal bovine serum (FBS; Invitrogen), 1000 U/ml of granulocyte-macrophage colony-stimulating factor (GM-CSF), and interleukin-4 (IL-4) (both from R&D) for 5 days. On days 2 and 5 of DC differentiation, culture medium containing GM-CSF and IL-4 was replaced, and on day 5, DC were matured by adding 100 ng/ml of lipopolysaccharide (LPS; SigmaAldrich). The remaining PBMC from the negative fraction of monocyte isolation were kept frozen. When needed, CD14-depleted PBMC were thawed, and CD4^+^ T cells were purified by negative immunomagnetic selection (Miltenyi Biotec). Cells were then cultured with RPMI containing 10% FBS without stimuli.

Monocytes, after isolation from PBMC, and mature DC (mDC), at day 7, were immunophenotyped by flow cytometry (FACSCalibur Flow Cytometer; BD Biosciences). The monoclonal antibodies (mAb) used for cell immunophenotyping were: CD14-FITC (clone M5E2; BD Pharmingen), DC-SIGN-PE (clone DCN45; BD Pharmingen), CD4-PerCP (clone SK3; BD Biosciences), HLA-A,B,C-PE (clone G46-2.6; BD Pharmingen), HLA-DR-PerCP (clone L243; BD Biosciences), CD86-FITC (clone 2331; BD Biosciences), CD83-PE (clone HB15e; BD Biosciences), and CD80-PE-Cy5 (clone L307.4; BD Pharmingen).

The T-lymphoblastoid MOLT-4/CCR5 (MOLT) cell line (National Institutes of Health [NIH] AIDS Research and Reference Reagent Program: from Dr. Masanori Baba, Dr. Hiroshi Miyake, Dr. Yuji Iizawa) [70] and the derivative cell lines chronically infected with X4-tropic (MOLT_NL4-3_) or R5-tropic (MOLT_BaL_) HIV-1 [71,72] were cultured in RPMI containing 10% FBS. TZM-bl (National Institutes of Health [NIH] AIDS Research and Reference Reagent Program: from J.C. Kappes and X.Wu, and from Tranzyme) and HEK-293 T (ATCC-LGC) cell lines were maintained in DMEM (Invitrogen) supplemented with 10% FBS. All media contained 100 U/ml penicillin and 100 μg/ml streptomycin (Invitrogen).

### Viral stocks and plasmids

Viral stocks of HIV_NL4-3_, HIV_NFN-SX_, Env-deficient HIV_NL4-3ΔENV_, and HIV_NL4-3Ren_ were generated by transfecting the proviral construct pNL4-3, pNFN-SX, pNL4-3Luc^+^Rev^–^Env-, and pNL4-3Ren, respectively, to HEK-293 T cells. HIV_NL4-3-Cherry_ was obtained following co-transfection of pCHIV and pCHIV mCherry in a 1:1 ratio [73]. The proviral vectors pNL4-3 and pNL4-3Luc^+^Rev^–^Env^–^ were obtained through the NIH AIDS Research and Reference Reagent Program, pNFN-SX[74] was kindly provided by Dr. W. A. O’Brien, and pNL4-3Ren[75], which contained the Renilla luciferase gene reporter, was a gift from Dr. S. Sánchez-Palomino. Briefly, HEK-293 T cells were transfected with calcium phosphate (CalPhos; Clontech) and supernatants containing virus were collected 48 hours later, filtered (Millex HV, 0.45 μm; Millipore), and frozen at −80°C until use. The HIV_NL4-3Ren_ stock was concentrated using the Lenti-X Concentrator kit (Clontech) after collection, according to the manufacturer’s instructions.

The p24^Gag^ content of all viral stocks was measured using an enzyme-linked immunosorbent assay kit (PerkinElmer). Titers of all viruses were determined using the TZM-bl reporter cell line [76]. Cells were assayed for luciferase activity 48 hours after infection (Bright-Glo Luciferase Assay System; Promega) in a Fluoroskan Ascent FL Luminometer.

### Measuring cellular conjugates

Cellular conjugates comprising uninfected or HIV-1-infected MOLT cells and primary target cells were quantified as previously described [20]. To analyze the formation of conjugates between mDC and autologous or allogeneic non-activated primary CD4^+^ T cells, we proceeded as follows. Purified CD4^+^ T cells were labeled with 15 μM of Orange CMRA fluorescent probe (Molecular Probes, Invitrogen) for 20 minutes at 37°C, washed twice with PBS and left overnight at 37°C in 5% CO_2_ in RPMI containing 10% FBS. Before performing the assays, labeled cells were washed again and resuspended in RPMI with 10% FBS. For those experiments performed in the presence of HIV-1, mDC were incubated with HIV_NL4-3_, HIV_NFN-SX_, or HIV_NL4-3ΔENV_ (50 ng p24^Gag^ per 1.5 × 10^5^ mDC) for 4 hours at 37°C in 5% CO_2_. To remove uncaptured viral particles, mDC were extensively washed with PBS. For blocking assays, mDC and CMRA-labeled CD4^+^ T cells were separately preincubated for 30 minutes at room temperature in the presence or absence of different mAb and reagents: α-LFA-1 (10 μg/ml, clone 68.5A5), α-ICAM-1 (10 μg/ml, clone RM3A5), α-ICAM-3 (10 μg/ml, clone 101.1D2) (all three kindly provided by Dr. R. Vilella, Hospital Clínic, Barcelona, Spain), α-CD4 Leu3a (10 μg/ml, clone SK3, BD Biosciences), isotype control antibody (10 μg/ml, eBioscience), staphylococcal enterotoxin A from *Staphylococcus aureus* (SEA) (10 μg/ml, SigmaAldrich), and cytochalasin D (5 μM, SigmaAldrich). Then, 75,000 mDC were co-cultured with 75,000 autologous or allogeneic CMRA^+^ CD4^+^ T cells for different incubation periods depending on the experiment (0 min, 30 min, 1 h, 2 h or 24 h) at 37°C in 5% CO_2_ in a final volume of 200 μl of RPMI containing 10% FBS on a 96-well flat-bottom plate, with and without shaking. Afterwards, 50 μl of formaldehyde 2% was added to the culture without perturbing cellular conjugates, and samples were analyzed in an LSR II flow cytometer equipped with a plate loader (BS Bioscience). All events with similar morphology to mDC (SSC) but simultaneously positive for the cell tracker CMRA were considered stable cellular conjugates of mDC and primary CD4^+^ T lymphocytes. Gating strategy for quantification of mDC-CD4^+^ T-cell conjugates is shown in Additional file 2 A. The percentage of cellular conjugates was calculated as follows: [conjugates/total CD4 CMRA^+^ cells]*100. Controls consisting of CMRA-labeled CD4^+^ T cells cultured alone were performed in each experiment to quantify the background levels of T-cell-T-cell conjugates, which was less than 0.01% (Additional file 2 B). Control co-cultures between DDAO-labeled mDC (CellTrace Far Red DDAO-SE, Molecular Proves, Invitrogen) and CMRA-labeled CD4^+^ T cells were performed to assess that the SSC-CMRA gating strategy unequivocally quantified mDC-CD4^+^ T-cell conjugates (Additional file 2 B). Similar percentages of mDC-CD4^+^ T-cell conjugates were obtained in both SSC-CMRA and DDAO-CMRA dot plot analyses.

### mDC-mediated HIV-1 *trans-*infection of non-activated primary CD4^±^ T cells

Transmission of HIV-1 from mDC to CD4^+^ T cells was assessed by co-culturing 1 × 10^5^ virus-pulsed mDC with 1.5 × 10^5^ autologous or allogeneic non-activated primary CD4^+^ T cells for 48 hours at 37°C in 5% CO_2_. First, mDC were incubated with HIV_NL4-3Ren_ at MOI = 0.1 (based on HIV-1 titration in TZM-bl cells) for 5 hours at 37°C in 5% CO_2_, and then cells were extensively washed with PBS to remove uncaptured viral particles. Subsequently, mDC and CD4^+^ T cells were separately pre-incubated for 30 minutes at room temperature in the presence or absence of the same mAb and reagents used to evaluate the cellular conjugates or with azidothymidine (AZT) (5 μM, NIH AIDS Research and Reference Reagent Program), or saquinavir (SQV) (0.5 μM, NIH AIDS Research and Reference Reagent Program). Then, HIV-1-pulsed mDC and autologous or allogeneic non-activated primary CD4^+^ T cells were co-cultured in a final volume of 200 μl of RPMI containing 10% FBS on a 96-well U-bottom plate in the presence of the indicated blocking reagents. After 48 hours of co-culture, luciferase activity was assayed with the Renilla-Glo Luciferase Assay System (Promega) using a 96-well plate Fluoroskan Ascent FL Luminometer. To specifically show the infection of CD4^+^ T cells in co-cultures, background values based on HIV-1-exposed mDC cultured alone were subtracted for each co-culture condition, although luminescence values of HIV-1-pulsed mDC were comparable to those of unpulsed mDC.

### mDC-mediated activation and proliferation of primary CD4^±^ T cells

Activation and proliferation of non-activated primary CD4^+^ T cells induced by mDC were analyzed after autologous or allogeneic co-culture. In brief, non-activated CD4^+^ T cells were stained with 0.35 μM of carboxyfluorescein diacetate succinimidyl ester (CFDA-SE) (Molecular Probes, Invitrogen) in PBS with 1% FBS for 6 minutes at room temperature, washed in PBS with 10% FBS and left for 30 minutes in RPMI containing 10% FBS at 37°C in 5% CO_2_. Cells were washed with PBS twice and finally resuspended in RPMI with 10% FBS. Then, 75,000 CFDA-SE-labeled CD4^+^ T cells were co-cultured with 75,000 autologous or allogeneic mDC at 37°C in 5% CO_2_ in a final volume of 250 μl of RPMI containing 10% FBS on a 96-well U-bottom plate. Previously, some mDC were incubated with HIV_NL4-3Ren_ at MOI = 0.1 (based on HIV-1 titration in TZM-bl cells) for 5 hours at 37°C in 5% CO_2_, and then cells were extensively washed with PBS to remove uncaptured viral particles. As positive controls, CD4^+^ T cells alone or with mDC were cultured in the presence of SEA or α-CD3 (clone OKT3, eBioscience). Negative controls based on CD4^+^ T cells cultured alone in the absence of any stimuli were used to set the basal levels of T-cell activation which were then used to analyze the flow cytometry data of mDC-CD4^+^ T cell co-cultures. After 16 hours or 5 days of co-culture, CD4^+^ T-cell activation and proliferation were evaluated by staining the cells with the following mAb: CD2-PerCP/Cy5.5 (clone RPA-2.10, BD Pharmingen), CD69-APC (clone FN50, BD Pharmingen), CD11c-APC/Cy7 (clone Bu15, BioLegend), and CD25 V450 (clone M-A251, BD Horizon). Cells within the CD2-positive CD11c-negative singlet gate were analyzed. Samples were acquired on an LSR II flow cytometer (BD Biosciences) and data were analyzed using FlowJo software (Tree Star) with a built-in proliferation platform.

### Confocal microscopy

mDC and non-activated primary CD4^+^ T cells were co-cultured for 2 hours at 37°C in 5 CO_2_. To unequivocally identify mDC and CD4^+^ T cells, co-cultures were stained with α-HLA-DR-AlexaFluor 647 (clone L243, BioLegend) and α-CD3-PE (clone HIT3a, BioLegend), respectively. To determine the polarization of the HIV-1-containing intracellular compartment in mDC to the contact zone between mDC and CD4^+^ T cells, mDC were previously pulsed with HIV_NL4-3Cherry_ for 4 hours, extensively washed, and co-cultured with CD4^+^ T cells. After co-culture, cells were fixed, cytospun onto glass slides and mounted with Fluoroshield with DAPI mounting medium (Sigma-Aldrich). Spinning disk confocal microscopy was performed on a PerkinElmer Ultraview ERS. Confocal Z-stacks were acquired at 0.25 μm steps using a 63× objective, and processed with Volocity 6.1 software (Improvision, PerkinElmer) using the maximum fluorescent intensity projection.

### Statistical analysis

Statistical analysis was performed using the Wilcoxon matched-pairs test in GraphPad software Prism v.5. p values <0.05 were considered statistically significant.

## Abbreviations

APC: Antigen-presenting cells; AZT: Azidothymidine; CFDA-SE: Carboxyfluorescein diacetate succinimidyl ester; DC: Dendritic cell; DC-SIGN: DC-specific ICAM-3-grabbing non-integrin; Env: Viral envelope glycoprotein; HIV-1: Human immunodeficiency virus type 1; ICAM-1: Intercellular adhesion molecule 1; ICAM-3: Intercellular adhesion molecule 3; LFA-1: Leukocyte function-associated antigen 1; LPS: Lipopolysaccharide; mAb: Monoclonal antibody; mDC: Mature dendritic cell; MLR: Mixed lymphocyte reaction; PBMC: Peripheral blood mononuclear cells; pMHC: Peptide-major histocompatibility complex; SEA: Superantigen; staphylococcal enterotoxin A from *Staphylococcus aureus*; SQV: Saquinavir; SSC: Side scatter chanel; TcR: T-cell receptor.

## Competing interests

The authors declare that they have no competing interests.

## Authors’ contributions

MTR-P designed and performed research, analyzed data, and wrote the manuscript; IP designed and performed research and analyzed data; NI-U, MCP, JC, and IE provided technical assistance and analyzed data; BC designed research; JB and JM-P designed research, analyzed data, and wrote the manuscript. All authors read and approved the final manuscript.

## Supplementary Material

Additional file 1 **A. mDC-mediated HIV-1 *trans-*infection experiments were designed at 48 hours to evaluate a single cycle of HIV-1 infection.** Control experiment consisting of SEA-treated mDC-CD4^+^ T-cell co-cultures in the presence of SQV confirmed that HIV-1 infection came from one cycle of viral replication, even in the presence of the SEA activating stimulus. RLU, relative light units. **B.** Evaluation of direct infection by free virus of non-activated primary CD4^+^ T cells and mDC cultured alone compared with HIV-1 mDC-mediated *trans*-infection to CD4^+^ T cells (autologous and allogeneic co-cultures). Cells were incubated with HIV_NL4-3Ren_ for 5 hours at 37°C in 5% CO_2_ at MOI = 0.1 (based on HIV-1 titration in TZM-bl cells) or equivalent effective MOI. Then, cells were washed to remove excess of HIV-1 and cultured in the presence or absence of α-CD3 (mAb OKT3) and SEA activation conditions. Infection was evaluated 48 hours after. RLU, relative light units. Click here for file

Additional file 2 **A. Gating strategy for quantification of mDC-CD4**^**+**^** T-cell conjugates by flow cytometry.** CD4^+^ T cells were CMRA-labeled and mDC were defined based on their morphology. Live cells were first gated in FSC-SSC dot plots to discard cell debris. Then, cellular conjugates were identified within this gate and quantified in a gate including CMRA positive events. Conjugates were those events with similar morphology to mDC (SSC) but simultaneously positive for the cell tracker CMRA coming from CD4^+^ T cells. Events corresponding to mDC are shown in orange, CMRA-labeled CD4^+^ T cells are shown in blue, and cellular conjugates between mDC and CD4^+^ T cells are in green. **B.** Quantification of background levels of T-cell-T-cell conjugates. Controls consisting of CMRA-labeled CD4^+^ T cells cultured alone yielded less than 0.01% of cellular conjugates, thus confirming that the gating strategy used for quantification of mDC-CD4^+^ T-cell conjugates did not consider two CD4^+^ T cells in contact. **C.** To confirm that the gating strategy shown in panel A (SSC-CMRA) unequivocally quantified mDC-CD4^+^ T-cell conjugates, control co-cultures between DDAO-labeled mDC and CMRA-labeled CD4^+^ T cells were performed. Co-cultures were analyzed by the gating strategy SSC-CMRA or considering conjugates as those events simultaneously positive for the cell tackers DDAO coming from mDC and CMRA coming from CD4^+^ T cells (DDAO-CMRA). Both quantification analyses yielded similar results. Click here for file
