# Supplementary figures and images for: The infectious synapse formed between mature dendritic cells and CD4+ T cells is independent of the presence of the HIV-1 envelope glycoprotein
Source: Retrovirology. 2013 Apr 16;10:42. doi: 10.1186/1742-4690-10-42 (PMC3640963; doi:10.1186/1742-4690-10-42)

# Additional File 1

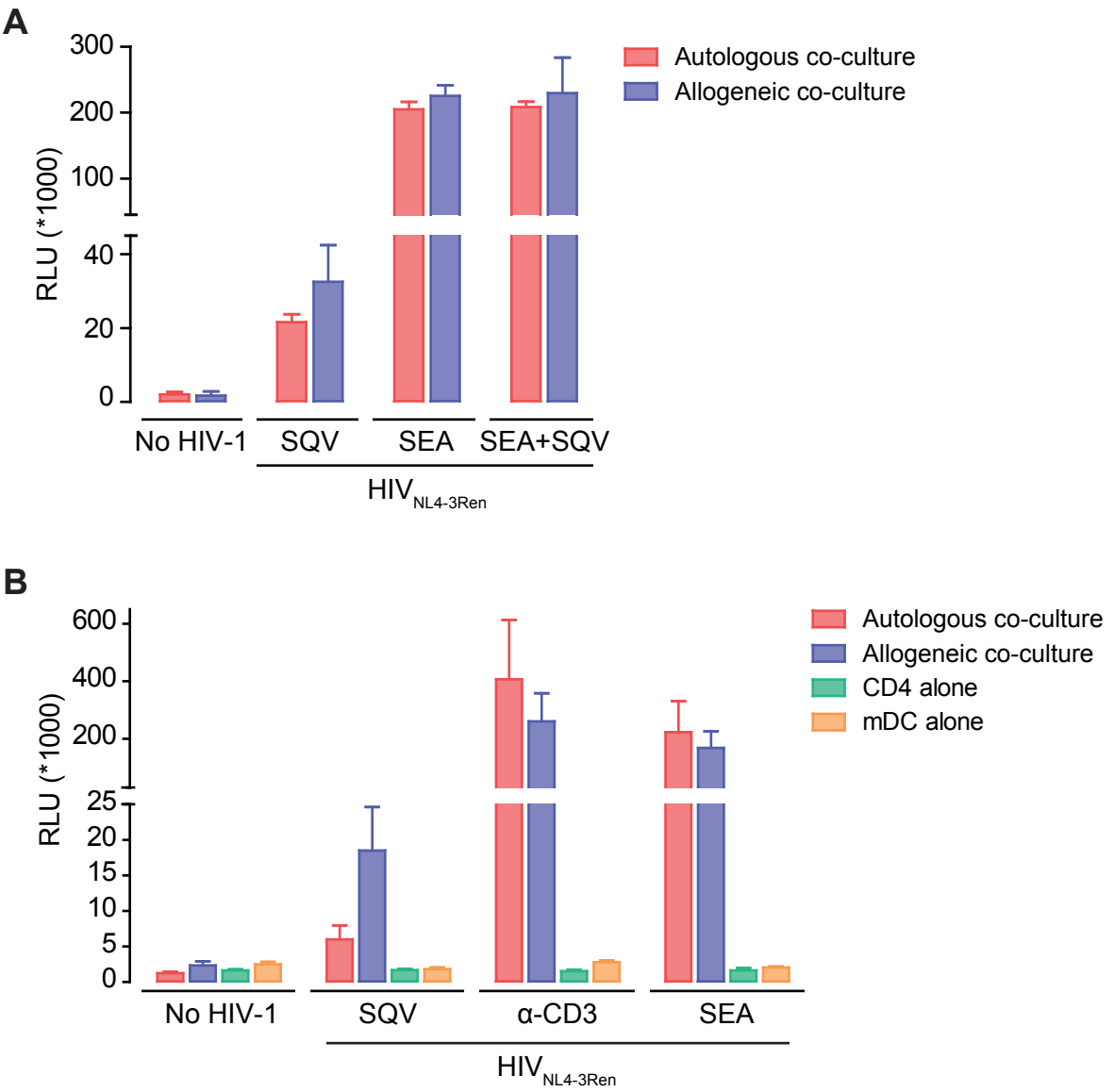

Supplement: Additional file 1 — A. mDC-mediated HIV-1 trans-infection experiments were designed at 48 hours to evaluate a single cycle of HIV-1 infection. Control experiment consisting of SEA-treated mDC-CD4+ T-cell co-cultures in the presence of SQV confirmed that HIV-1 infection came from one cycle of viral replication, even in the presence of the SEA activating stimulus. RLU, relative light units. B. Evaluation of direct infection by free virus of non-activated primary CD4+ T cells and mDC cultured alone compared with HIV-1 mDC-mediated trans-infection to CD4+ T cells (autologous and allogeneic co-cultures). Cells were incubated with HIVNL4-3Ren for 5 hours at 37°C in 5% CO2 at MOI = 0.1 (based on HIV-1 titration in TZM-bl cells) or equivalent effective MOI. Then, cells were washed to remove excess of HIV-1 and cultured in the presence or absence of α-CD3 (mAb OKT3) and SEA activation conditions. Infection was evaluated 48 hours after. RLU, relative light units. [file 1742-4690-10-42-S1.pdf]

## Additional File 2

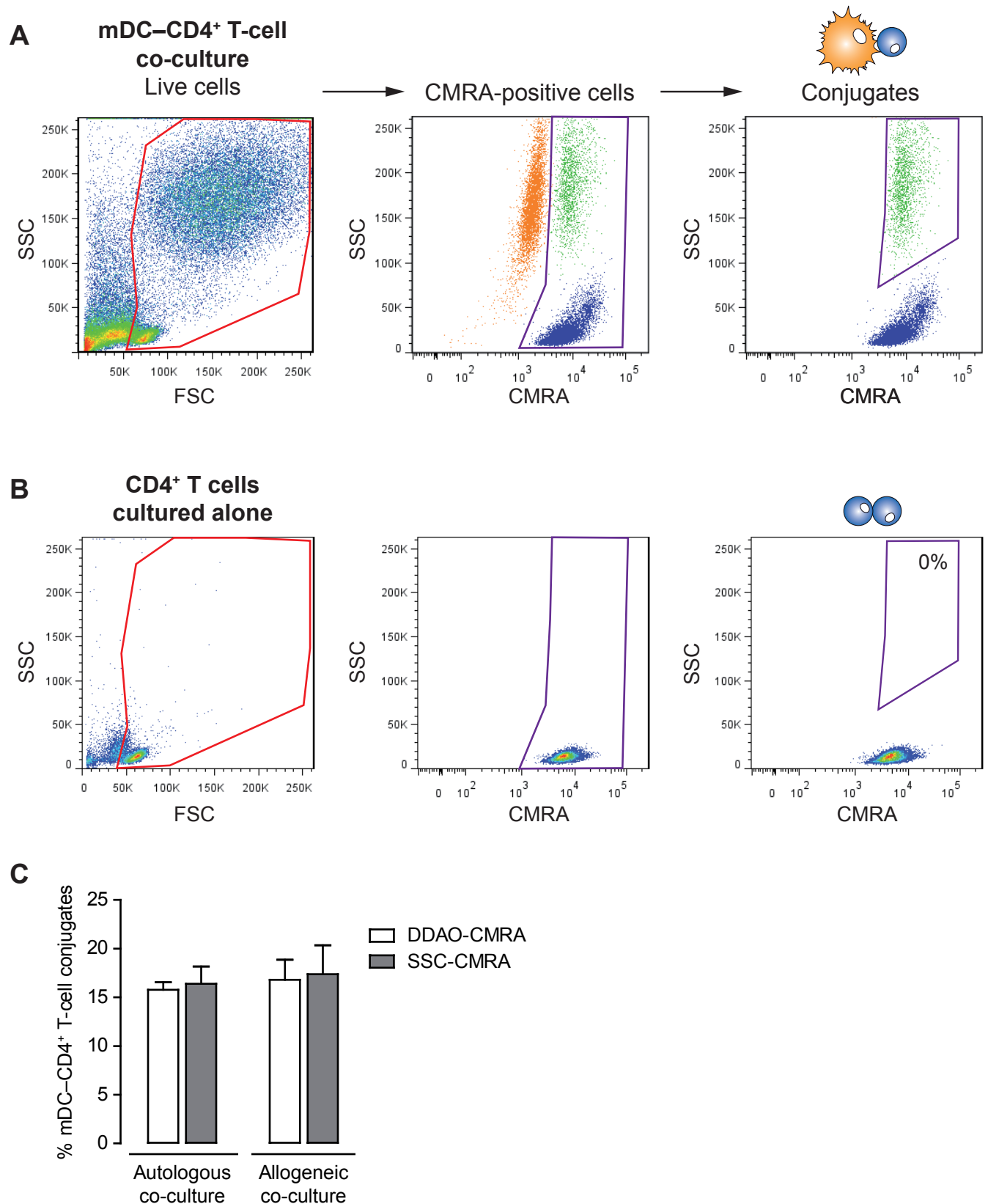

Supplement: Additional file 2 — A. Gating strategy for quantification of mDC-CD4+ T-cell conjugates by flow cytometry. CD4+ T cells were CMRA-labeled and mDC were defined based on their morphology. Live cells were first gated in FSC-SSC dot plots to discard cell debris. Then, cellular conjugates were identified within this gate and quantified in a gate including CMRA positive events. Conjugates were those events with similar morphology to mDC (SSC) but simultaneously positive for the cell tracker CMRA coming from CD4+ T cells. Events corresponding to mDC are shown in orange, CMRA-labeled CD4+ T cells are shown in blue, and cellular conjugates between mDC and CD4+ T cells are in green. B. Quantification of background levels of T-cell-T-cell conjugates. Controls consisting of CMRA-labeled CD4+ T cells cultured alone yielded less than 0.01% of cellular conjugates, thus confirming that the gating strategy used for quantification of mDC-CD4+ T-cell conjugates did not consider two CD4+ T cells in contact. C. To confirm that the gating strategy shown in panel A (SSC-CMRA) unequivocally quantified mDC-CD4+ T-cell conjugates, control co-cultures between DDAO-labeled mDC and CMRA-labeled CD4+ T cells were performed. Co-cultures were analyzed by the gating strategy SSC-CMRA or considering conjugates as those events simultaneously positive for the cell tackers DDAO coming from mDC and CMRA coming from CD4+ T cells (DDAO-CMRA). Both quantification analyses yielded similar results. [file 1742-4690-10-42-S2.pdf]
